# Social determinants and mental health needs of Palestine refugees and UNRWA responses in Gaza during the COVID-19 pandemic: a qualitative assessment

**DOI:** 10.1186/s12889-022-14771-9

**Published:** 2022-12-08

**Authors:** Zeina Jamal, Zoheir ElKhatib, Shatha AlBaik, Masako Horino, Mohammed Waleed, Farah Fawaz, Giulia Loffreda, Akihiro Seita, Sophie Witter, Karin Diaconu

**Affiliations:** 1 Institute for Global Health and Development, Queen Margaret University, Musselburgh EH21 6UU Edinburgh, Scotland, UK; 2 The United Nations for the Relief and Works Agency in the Near East (UNRWA) Field Office, Gaza, Palestine; 3 The United Nations for the Relief and Works Agency in the Near East (UNRWA) Headquarters, Amman, Jordan; 4 Center for Human Nutrition and Sight & Life Global Nutrition Research Institute, Dept of Int’l Health, Johns Hopkins Bloomberg School of Public Health, Baltimore, MD USA

**Keywords:** Mental health, Social determinants, COVID-19, Pandemic, Refugees, Occupied Palestinian territory, Gaza, UNRWA

## Abstract

**Background:**

Due to pre-existing difficulties, refugees are especially susceptible to the negative effects of the pandemic; nonetheless, the pandemic’s effect on this group is still unclear. The purpose of this study was to determine the effects of the COVID-19 pandemic on the mental health of Palestine refugees in Gaza by identifying the role of social determinants. During the pandemic, the United Nations Relief and Works Agency for Palestine Refugees in the Near East (UNRWA) enacted a number of policies and measures. The purpose of this research was to assess their efficacy and acceptability.

**Methods:**

This qualitative study took place between August and November 2020. Twenty-nine key-informant interviews were conducted remotely with UNRWA Headquarters, field and clinical staff in Gaza and with community members, aged ≥18 years and residing in Rafah and Jabalia camps. We sought informed consent verbally or via email. Data was coded based on the framework for social determinants of mental health.

**Results:**

Interview results indicated that the relationship might be unidirectional, with COVID-19 causing the degradation of living conditions and vice versa, with living conditions exacerbating the COVID-19 situation by facilitating virus transmission. In other instances, the association between mental health determinants and COVID-19 might be bidirectional. In terms of experiencing violence and anxieties, women, children, and daily-paid employees were significantly more disadvantaged than other groups in the community. UNRWA modified its service delivery techniques in order to continue providing essential services. In general, UNRWA’s strategies throughout the pandemic were deemed beneficial, but insufficient to meet the needs of Gazans.

**Conclusion:**

The pandemic highlights the need to go beyond disease treatment and prevention to address social determinants to improve refugees’ health and reduce their susceptibility to future shocks. UNRWA has rapidly implemented telemedicine and mental telehealth services, making it imperative to assess the efficacy of these novel approaches to provide care at a distance. A long-term option may be to employ a hybrid strategy, which combines online and in-person therapy.

**Supplementary Information:**

The online version contains supplementary material available at 10.1186/s12889-022-14771-9.

## Introduction

### Background

The Arab-Israeli war officially started with the Nakba and the founding of Israel on May 15, 1948. By 1948, the Jewish forces had systematically depopulated 500–600 Palestinian villages and/or cities; threatening the lives of Palestinians, permanently expelling and sending into exile about 750,000 Palestinians [1]. By mid-2021, the population of Palestine refugees has grown to reach 13.8 million, scattered around the world [2]. Following the United Nations Partition Plan in 1948, Gaza city, and the area surrounding it, was allocated to the Arabs. Since then, this area became populated by internally-displaced Palestinians that were living in poverty in impoverished camps and were later offered aid by the United Nations for the Relief and Works Agency in the Near East (UNRWA) starting 1949 [3]. Since its establishment in 1949, UNRWA has been mandated to provide assistance to Palestine refugees in Syria, Lebanon, Jordan, Gaza and the West Bank (including East Jerusalem). In addition to providing health care, UNRWA provides education, relief and social services, microfinance and emergency assistance [4].

Between 1948 and 1967, the Gaza Strip witnessed several armed conflicts between Israeli forces and Arab guerrillas fighting against Israel; it was taken by Israel the first time in 1956 and reverted back to Egyptian control a year later, and was occupied again by Israelis in June 1967 after the Six-Day War that took place back then. The territory remained occupied by Israelis from 1967 to 2005 after the first and the second Intifada, which took place in 1987 and 2000, respectively, that forced Israeli troops to gradually retreat and transfer governmental control to the Palestinian Authority (PA) [3]. During the occupation of the Gaza Strip, the Israeli forces limited the movement of people and the exchange of commodities with other parts of Palestine. It restricted access to land and sea borders resulting in weak health and social services [5]. After the PA took control over the Strip, violence escalated between different Palestinian political groups, which led to the transfer of power in Gaza to Hamas in 2007. Hamas, listed as a terrorist group by Israel, the United States and the European Union, brought sanctions onto the Strip [3]. Since 2007, Israel, followed by Egypt, have enforced a blockade on the Gaza strip, thus crippling the lives of Palestinians even further and depriving its inhabitants from basic commodities such as fuel, food, and medicine. Since the blockade, Gaza was subjected to four major military operations by Israeli troops, with the last taking place in August 2022 and lasting for 3 days [6].

Approximately, 1.95 million Palestinians reside inside the 365 km^2^ area of the Gaza strip, of whom 1.47 million are registered refugees with UNRWA whereby 37.1% are living in 8 highly populated camps [4], compared to 871,000 registered refugees in the West Bank who live in 19 camps. This profile emphasizes the highly populated, scarce-resource setting of the Gaza strip [7]. Palestine refugees living in Gaza, especially those living in camps are at the bottom of the socioeconomic ladder [8]. In 2019, a year before community transmission of COVID-19 in Gaza, the level of unemployment was surging high reaching 45.1% [9]. Estimates of the same year by UNRWA show that 620,000 Gazans survive on $1.60 per day and nearly 390,000 Gazans live in absolute poverty [10]. The blockade, the dire socioeconomic status and the frequent exposure to violence have pushed Palestinians further into despair, with negative impacts on mental health and wellbeing [5].

Gaza has one of the youngest populations globally, with 64.2% of the population in 2021 being under 25 [11, 12]. The majority of studies on anxiety and depression in Gaza were conducted on children and adolescents [13–15]. A study in 2008, which included adult participants, was conducted on 200 parents and 197 children (aged 9–18 years) living in areas subjected to ongoing shelling and other military violence in Gaza. This study showed high rates of post-traumatic stress disorder (PTSD) and anxiety in both groups, with exposure to war trauma significantly deteriorating the mental health of both parents and their children. In this study, 77% of children were likely to present with PTSD using the Children’s Revised Impact of Events Scale (CRIES-13) cut-off score of 30 and 60% of parents were likely to have PTSD with potential clinical significance as assessed using the PTSD-Checklist with a cut-off of 50 [16]. Similar results around PTSD rates amongst adolescents were published a year earlier. Data from 2007 showed that 68.9% of adolescents had PTSD, 40% had moderate to severe levels of depression and 94.9% had severe anxiety [8]. According to the Lancet Commission on Global Mental Health, the origin of most of mental disorders go back to childhood and adolescence [17]. A review in 2015 showed that the prevalence of PTSD among children after a single episode of assaultive violence in a high-income context, such as that reported after being subjected to a mass shooting in the United States, varies between 8 and 91%. This huge variation raises methodological concerns such as the scale and diagnostic criteria used, the pre-incident psychological status and the timing of which the assessment took place as the prevalence of psychiatric disorders usually decreases over time [18]. For these reasons, it is difficult to compare prevalence estimates between different studies, using different diagnostic tools, and after varying time points post-event. Nonetheless, in 2016, data from UNRWA on protection and related services showed that the prevalence of depression and PTSD amongst Palestine refugees in general was greater than global figures [19].

In 2012, the United Nations published a report on the future of Gaza warning that by 2020 the territory will not be a liveable place if no remedial action to end the siege is taken [20]. Since the siege did not end, Palestinians living in Gaza continue to face multi-faceted psychosocial vulnerabilities, which the SARS-CoV-2 pandemic is likely to exacerbate. Countries and settings that were already experiencing a humanitarian crisis are particularly affected by the negative medical, economic, and psychological aspects of the pandemic [21]. The deterioration of mental health of different population groups during the COVID-19 pandemic has been documented worldwide [22]. According to the United Nations High Commissioner for Refugees (UNHCR), the pandemic itself and its associated mitigation and prevention measures has added to the deterioration of the mental health status of refugees [23].

Global evidence suggests that mental health disorders in populations worldwide are socially determined to a great extent [24, 25]. Social determinants of mental health are defined as the driving force that shapes wellbeing at the individual and community levels. They are the social, economic and environmental factors that affect the onset and prognosis of mental health illnesses. They also increase the risk for physical illnesses and worsen the patients’ prospects [26]. In 2018, the Lancet Commission on Global Mental Health and Sustainable Development provided a new conceptualization of social determinants of mental health, bringing together distal and proximal factors and associating them with the Sustainable Development Goals (SDGs) in an attempt to drive the reduction of global mental health while progressing towards the attainment of SDGs.

As the refugee population in Gaza is already facing a convergence of stressors, the detrimental effects of the pandemic on mental health and its social determinants amongst this vulnerable group is important to explore. Specifically in this research, we aimed to explore the impact of the COVID-19 pandemic on the social factors and daily living conditions affecting the mental health of Gazans living in Rafah and Jabalia camps, and to document views around the effectiveness of UNRWA’s policies and services that were enacted by UNRWA during this stressful period, in order to address the vulnerabilities of the study population and design targeted interventions.

## Methods overview

This was a qualitative study and the data in this paper is a subset of a bigger dataset obtained in another study that took place in Gaza and Lebanon’s fields of operations on the effectiveness, equity, acceptability, and scalability of the strategies enacted by UNRWA during the COVID-19 pandemic, and underlying health system and community capacities supporting strategy implementation [27]. In the overall research, interview questions explored contextual differences between Lebanon and Gaza in relation to COVID-19 response strategies and the arrangements made to ensure continuity of services (with a particular attention to mental health and non-communicable diseases (NCDs) services). Also, interviewees were prompted to reflect on the stressors experienced and coping strategies adopted during the pandemic (Supplementary material-1). The overall sample size of the original dataset is 45 key-informant interviews across Gaza and Lebanon. At the time of data collection, Palestine refugees in Lebanon were facing a multi-layered crisis, compounding the pandemic woes. The political unrest, economic collapse and the Beirut blast on August 4, 2020 created a different set of challenges for Palestine refugees in Lebanon and prompted a different response from UNRWA. For this reason, we restricted our scope in the current manuscript to data extracted from 29 key-informant interviews of UNRWA- Headquarters (HQ), staff, and community leaders/ members based in Gaza only.

### Key-informant interviews

#### Participants and sampling

ZJ carried out interviews with UNRWA HQ, Gaza Field Office, Health and Relief and Social Service UNRWA staff and with community leaders/ members residing in Jabalia and Rafah camps. Inclusion and exclusion criteria are detailed in Table 1. The reason why these camps were selected is because they are the most densely populated refugee camps in Gaza and are geographically dispersed across the Strip. The sampling strategy was guided by principles of data saturation and diversification. The latter was important to reflect various views, levels of knowledge and experience. Determining the sample size needed to reach data saturation in qualitative research is largely debated in the literature with recommendations around recruitment starting with a minimum of 15 participants [28] to between 30 and 60 participants by others [29]. In the current study, participants were selected using purposive, convenience and snowball sampling.

**Table 1:** Participants’ inclusion criteria

Participant category	Eligibility criteria
UNRWA staff	a) actors in the Health Programme and in the Relief and Social Services Programme engaged in routine and/or COVID-19 related service delivery at the clinical, area, field, or the headquarter level b) in position for 1 year or more and c) ≥18 years old
Community members/ leaders	a) Palestine refugees i.e. registered with UNRWA b) residents of either Rafah or Jabalia camps c) ≥18 years old

#### Recruitment

The Research Coordinator at UNRWA (SA) was in charge of recruiting key-informants (UNRWA staff and community leaders). SA contacted all potential key informants working at UNRWA explaining the study and inviting them to reply to the study team if they wish to participate. Also, SA contacted community leaders at each camp, explained what the study is about and explained all procedures of informed consent including the voluntary participation, the possibility to withdraw at any point and the absence of any benefit or harm from UNRWA for those who wish to participate or not. In order to avoid any feeling of coercion, SA told community leaders that they can confirm whether they would like to participate or not in a subsequent call. ZJ contacted each participant who agreed to participate to inform him/her about the study and seek the participant’s consent. Upon giving consent, ZJ agreed with each participant on a date/time and means for carrying out the interview. The interviews were carried out for 3 months starting August 2020. Details of recruited participants are outlined in Table 2.

**Table 2:** Participants of Key-Informant Interviews

Participant category	Participant details	Number
UNRWA Headquarters (HQ) staff	Members of the COVID-19 Coordination Body (e.g. representatives from Protection, Planning, Security and Risk Management (SRM) among others) who were responsible for the overarching coordination and core administrative functions	4
Field and area level^a^	Staff at the health, social work, planning, protection, operations, and emergency departments in Gaza Field Office	9
Health and social care professionals (camp-based) ^a^	Doctors (general or specialist), Nurses, Pharmacists, Head of health centers and coordinators, Social and relief service workers, and Psychosocial counsellors at either Rafah or Jabalia health centers	12
Community members/ leaders	Community leaders (e.g., political, religious or leaders of NGOs) and members	4

^a^Participants recruited from the Gaza Field Office and those working at the health centres were responsible for context specific operations and for ensuring the service delivery and continuity of health and social and relief services at Jabalia and Rafah camps

#### Data collection

All interviews were conducted remotely by the researcher ZJ via Microsoft Teams(MS-Teams) or WhatsApp. Interviews were conducted either in Arabic or English (upon participant’s preference) and lasted 45 minutes on average. They were audio-recorded upon the participant’s consent, translated, and transcribed, verbatim and at the same time, directly to English. The transcription was carried out by the field researcher MW and FF and were verified by ZJ. Participants utilised their own phones or laptops when interviewed. The interviews were carried out in a private place that the participant was comfortable at which was either at their office or their place of residence.

Interviews with UNRWA staff members (at HQ, field, area and camp levels) addressed the policies and services that were enacted by UNRWA during the COVID-19 pandemic, the impact of the pandemic and the containment measures adopted by UNRWA on the Palestinian community, the arrangements to continue routine service delivery, including mental health and psycho-social support (MHPSS) and NCD services and the stressors and coping mechanisms practiced by staff during the pandemic. Interviews with community members focused on identifying community stressors and enacted coping strategies, trust in UNRWA and acceptability of the COVID-19 related health response, and ability and willingness to act on public health advice. Interview questions were developed by the study team to answer the research questions of the study. Before starting data collection, a pilot interview with an UNRWA staff was carried out to ensure clarity of the questions. The interview guide for the different participant groups is found in Supplementary material-1.

#### Analysis

**Fig. 1:**
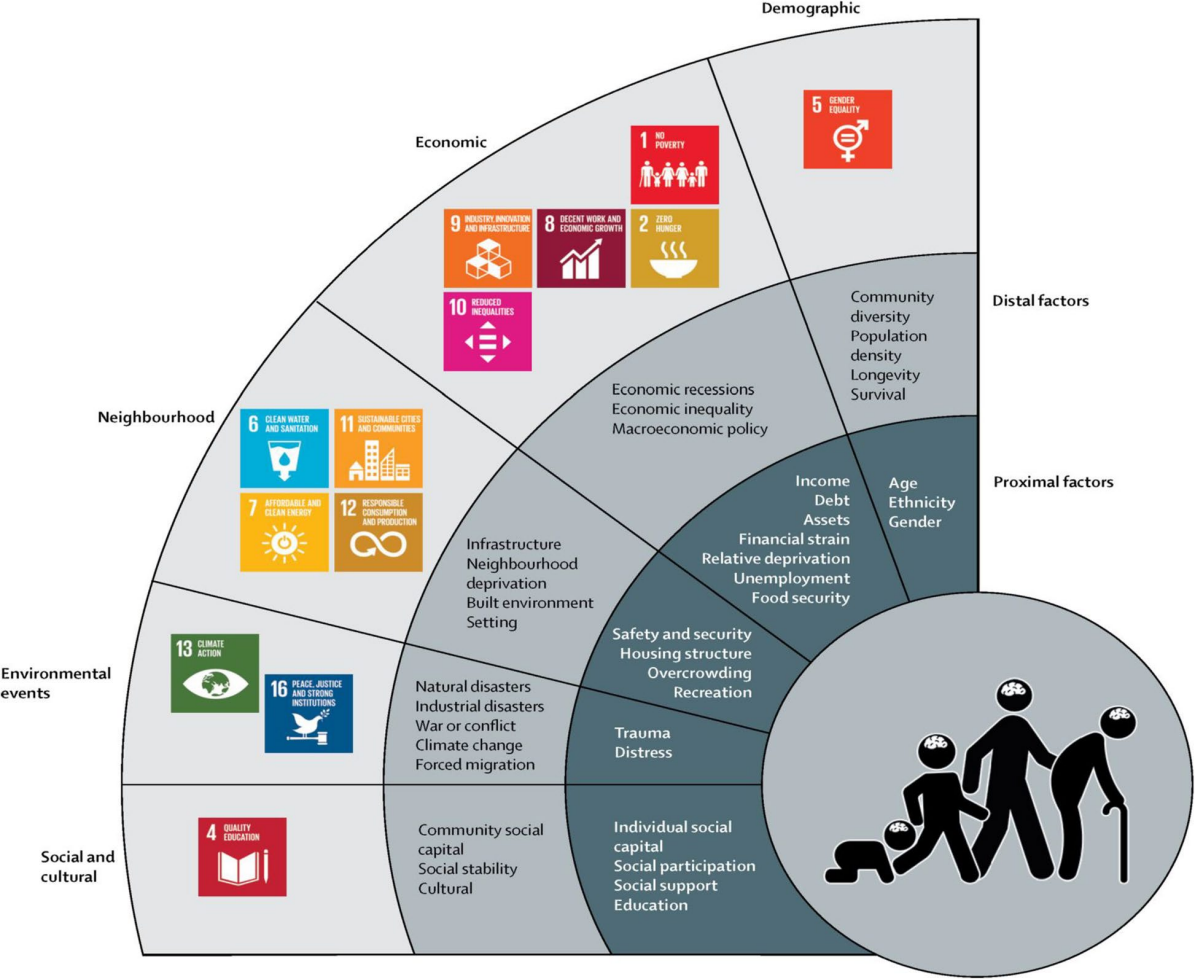
Social Determinants of Mental Health and the SDGs [17, 31]

Data was analysed using thematic content analysis, using a combination of inductive and deductive approaches. The analysis of interview data was done following the outlined steps:

- Becoming familiar with the dataset: This phase involved reading and re-reading the transcripts and sometimes going back to the original audio-recordings. The purpose of this phase was to become immersed and deeply familiar with the information presented in the interviews.
- Coding of the interviews: Interviews were uploaded on Dedoose Version 7.0.23 for coding and subsequent analysis [30]. ZJ coded the interviews, and held several deliberations with KD to finalise coding tree and add new codes when relevant. The codes were developed to cover the elements of the conceptual framework on Social and Cultural determinants of Mental Disorders and the Sustainable Development Goals (Fig. 1). An additional code and subcodes were added to answer the second research question on UNRWA’s strategies. Using an inductive approach, information on UNRWA’s strategies were extracted.
- Generating themes and presenting the analytic narrative: Initially, ZJ started collating related codes and began developing initial themes around them. After that, ZJ started reviewing the generated themes, looking for patterns and overlap and weaving together the narrative so that the final list of identified themes genuinely “tell the story” behind the data. This whole process was iterative as ZJ was going back and forth to the raw data for deeper understanding and reflection and to collate relevant excerpts that illustrate the ideas presented.

## Results

The analysis of the 29 interviews identified four major themes and 9 subthemes. The first three themes fit well with the proximal and distal factors outlined earlier in the framework on social determinants of mental health (Fig. 1). They describe the relationship between COVID-19 and the determinants of mental health. Evidence from interview data shows that the relationship could be one way, with COVID-19 leading to the deterioration of living conditions, and vice-versa i.e. with living conditions worsening the COVID-19 situation by favouring virus transmission. In other situations, the relationship between determinants of mental health and COVID-19 could be bidirectional. For instance, unemployment and financial strain were negatively impacted by the pandemic and breaking lockdown measures to secure income favoured virus transmission. Theme 4 discussed the services and strategies that UNRWA enacted in response to the pandemic and outlines the community’s views around those strategies, in terms of effectiveness and acceptability.

### Theme 1: determinants which deteriorated as a result of COVID-19

Participants’ experiences during the pandemic illustrate how this health crisis have worsened their mental wellbeing by negatively impacting the conditions in which they live in. Violence at the community level and reported cases of Gender Based Violence (GBV), especially against women and girls, increased during the pandemic; both of which were attributed to the lockdown measures. Limited resources during the pandemic meant also that households had increased food insecurity.

- Violence

Precautionary measures to curb the spread of COVID-19 pandemic instigated episodes of violence between the public and the police. As the police were trying to enforce lockdowns, they often clashed with the people as they were trying to break the forced confinement.

*“There were conflicts between people and the police for not complying with the instructions due to COVID… people were fed up and they didn’t abide by the instructions, which caused conflicts with the police. Frankly speaking, unhealthy meals were provided to patients at the governmental isolation areas. Also, if there was an infection, the authorities will quarantine the whole building and keep everyone locked up, without providing them with their food need, so people ran away. People were suffering both ways, whether quarantined at their homes or at the government isolation centres... In short, the pandemic affected three aspects: social, economic and psychological.” Psychosocial counselor_Jabalia 4*

b) Gender-based-violence

Interview data with UNRWA HQ staff suggest that GBV, particularly violence against women and girls, became more common during the pandemic, mainly due to home confinement and forced lockdowns.

*“Data indicates that we have an increase in domestic violence related to abuse within the family particularly on females. I think that is also affecting the mental health during the pandemic”. Headquarter staff_1.*

*“Of course, home quarantine generates a lot of family violence as a result of psychological pressure on people. Home quarantine is not easy especially when it is obligatory”. RSS_Jabalia 3*

iii) Food insecurity

The COVID-19 pandemic increased the number of people in Gaza suffering from food insecurity. Reduced income impacted Gazan’s ability to buy foods and UNRWA’s in-kind assistance was delayed for those eligible and was only given to a segment of the society while in reality, the majority of people were in need of such aid.

*“Definitely this pandemic has affected food security and as I mentioned before, the majority of the Palestinian people live on an irregular daily work. A person for example, cannot leave his job, stay at home and deprive his family from many basic needs just to abide by the protective measures against coronavirus. The economic situation is very difficult for many people”. RSS-Jabalia 2.*

*“People stuck at home due to Coronavirus and this in itself has affected people negatively, as they don’t have enough food. I am talking about the majority (almost two-thirds of the community).” Psychologist_Jabalia 4.*

Food insecurity impacted people who were under forced confinement at home as there was no contingency plan to regularly provide food for such families. Even those who were quarantined at hotels and isolation centres complained about the food, saying that the food served was not fresh nor was it nutritious.

*“People were quarantined for a week and no one asked about them. They were trying to call the free number, but there was no answer… they [UNRWA staff] answered their calls, but after 10 days, then they brought food and aid. They provide aid to the quarantined, but frankly, irregularly. A large family needs to eat every day and only one meal is not enough. For example, my sister was quarantined, the first day they brought her rice, the second day they brought her cheese and milk, but what about the children? Where is the bread, where is the flour, where is the milk? Third and fourth day they did not bring anything, and so on”. Community member_Jabalia 1*

iv) Accessing education

The education process got disrupted during the pandemic. Remote learning requires proper resources and infrastructure to carry it on. Interviewees said that UNRWA schools were offering remote learning as schools were closed most of the year 2020. However, not all students were able to access remote learning in a resource-scarce setting such as Gaza. First, the internet connection is very bad and parents cannot afford to buy laptops or iPads for their children.

*“I was hoping UNRWA would do more, but you know in Gaza, we have limited resources. However, at least, to provide students with school books, how students can study without having textbooks. Okay they are available on the internet, but in Gaza, not all people have mobiles, iPads or laptops. Also, the distant learning curriculum is very difficult and intense and not all parents are educated. UNRWA currently started to distribute school books to parents, as there’s nothing more they can do.” Psycho-social worker_Rafah 5.*

In addition, a school principal in Jabalia mentioned also that some parents do not have smart phones which could have been a replacement in the absence of computers and other expensive devices.

Interviewed MHPSS staff noted that children showed elevated levels of aggression because they were confined at home with limited opportunities to learn and play.

*“This pandemic has greatly affected children and they were showing very violent behaviours without control. There were no schools, no electricity, no technology, and not all parents are educated to help their children with distance learning”. Psychosocial counselor_Jabalia 4.*

*“Parents also have noticed negative behaviours among their children as schools and streets were the only breathing space for them. I always recommend people to spend more time with their children, engaging them in doing useful things using simple materials” Psycho-social worker_Rafah 5*

e) Social support, community ties and good work relationships

Interview data draw on examples of social capital or community ties that were displayed during the pandemic. For community members, UNRWA is perceived as a pillar of stability that provides necessary services and support for Palestine refugees in Gaza.

*“UNRWA is considered as a pillar... our lives depend on UNRWA and the services it provides whether it is health services, coupons for food, education etc. We rely on UNRWA in everything. We fully trust UNRWA even now during the pandemic” Community member- Jabalia 1.*

Within UNRWA, the solidarity that the staff have shown and the cooperation between different stakeholders to act collectively to serve the Palestinian community are examples of social capital.

“*Working from home is not an easy job and it is a big challenge for me and my family to provide the services required for that, in addition to the many tasks, we were asked to achieve… we serve our people, this is considered a patriotic and religious duty.” Social Worker_Jabalia 3.*

*“There was coordination between some associations and UNRWA. Some NGOs provided quantities of food parcels and medicines… and this is what I saw in reality”. Community member_ Jabalia 1.*

*“…We currently work in cooperation with the ministry of health and some NGOs. We have a list of NGOs with their contact details and addresses. If we detect a critical case over phone, we contact the NGOs and give them the case’s address and contact details and explain to them the exact condition of such case in order to make a home visit to them” Physician_Rafah 1.*

Interview data show that one of the stressors that people went through during the pandemic was the fact that physical and social distancing separated people from their social support network. The concept of physical and social distancing is foreign for Palestinians as they usually draw on the social support of friends and family to overcome emergencies.

*“We are a conservative society, as family relations are great, but the COVID-19 pandemic was a shock to the Palestinian community. New concepts were introduced to people that were not desirable for them, such as social distancing, not shaking hands, wearing masks and gloves on a daily basis, preventing weddings, condolences and praying in mosques, many things. But we must change our perceptions and behaviours in order to protect ourselves from this disease.” Community members_Rafah 2.*

One interviewee said that social support remained during the pandemic. COVID-19 prevention measures did not prevent people from helping each other.

*“… neighbours help too. There is a family interdependence among people, even if UNRWA does not afford aid, people help each other.” Community member_Jabalia 1.*

However, not all participants agreed on that. UNRWA staff had the impression that the social stigma against COVID-19 patients was common in Gaza. The stigma was reinforced through malpractices by the health authorities when dealing with a newly identified case, the spread of fake news and misinformation and the day-to-day interactions between members of the community. As a result, stigma has created health inequalities, reduced social interactions and support during the pandemic.

*“I think there is a stigma in the community due to a number of mistakes made when dealing with cases by local authorities… when someone is tested positive for COVID-19, the local authorities go to pick him/her with an ambulance and a police car, so you can hear the ambulance sirens all the way to them and they also take their family members to be isolated in isolation centres. Accordingly, this caused a very negative reaction from the community and looked like it is a charge to be infected with COVID-19”. Gaza Field Office staff 5.*

*“Honestly, yes, they’re suffering from stigma, and that’s putting psychological pressure on them. People tend to isolate the infected people with COVID-19 and they are afraid to make any contact with them even after they recover”. Psychosocial counselor_Jabalia 4.*

Stigma related to mental health illnesses was perceived by psychosocial workers at UNRWA to have reduced in the past few years. People with poor wellbeing seem to be seeking professional help more than before. During the pandemic, UNRWA staff had the perception that people were more accepting to receiving psychological help should they need it.

*“Previously, people felt stigma to have mental issues or refused to go to psychiatrists, but nowadays people accept the situation and doctors were transferring patients to the psychiatrist clinics without any problems”. RSS_Rafah 5.*

At the level of UNRWA, staff supported each other very much during the pandemic. The support received from colleagues and from the higher administration boosted the staff morale and enhanced their job performance.

*“…It [the pandemic] made them [UNRWA staff] closer to each other and they shared their concerns. I did a lot of things to support the staff “you are great“, psychodrama and a breakfast gathering. I think that the relationship between the staff is now way better than before, as they have time to sit and talk to each other. UNRWA’s staff members were also exchanging roles aiming at providing the service.” Psychosocial counselor_Jabalia 4.*

*“Moral support yes and this support really gave us a big push forward to continue working. Our administration at first announced if anyone of you is sick or afraid of getting sick from this disease, he/she may not work and can simply stay at home. But we insisted on working, since this is a humanitarian act that we cannot leave. The administration was very supportive as the line manager and the director of our department were in contact with us. Especially our department manager who was constantly communicating with us, and this indicates his sincerity at work. Personally, when I spoke to him one day and informed him that my car at work was broken and I could not work in this way, he understood and responded to me in an hour as he lent me his car which he uses for work and gave it to me so that I could continue my work to the fullest.” RSS_Jabalia 3.*

### Theme 2: determinants which aggravated COVID-19

Interviewed participants mentioned how the Gaza strip is densely populated and houses are overcrowded. Also, several interviewees talked about the lack of proper services and infrastructure within the Strip. Lack of ventilation, poor lighting, unreliable electrical supply, and poor internet connectivity were examples of environmental conditions that were mentioned in the interviews to affect people’s wellbeing and health in general.

Interviewed UNRWA staff mentioned that over-crowdedness favours virus transmission, worsens mental health, especially during lockdown episodes, and poses a health risk for elderly and NCD patients who cannot distance themselves away from other people.

*“It [COVID-19] will be spread in a very fast way because of the highly populated areas, we are talking about the camps. I don’t know if you know how it is in the refugee camps in Gaza, it is very crowded. And if we are talking about physical distancing, it will not be possible at all. Even in the same house, you will find 10- 12 persons living in a small space”. Gaza Field Office staff 3.*

*“We have an abnormal population density in Gaza Strip. For example, we work in camps, and houses are very narrow, in one house there are ten or eleven people, or more, so imagine how difficult to quarantine people in houses like these. Thus, you find people standing at the doors of their houses. The houses are bad and there is not enough electricity, but anyway this is the bitter reality” RSS- Rafah 3.*

*“The houses are small and in poor condition, there is no ventilation or electricity”. Gaza Field Office staff 4.*

These hard living conditions were also reported by UNRWA staff, especially females, as an obstacle to carry out work-related tasks and a source of stress. Female staff members reported challenges related to juggling their work with housework, childcare and attending to family needs while performing work-related tasks using unreliable internet connection.

*“It greatly affected me to stay at home, because I have small children and sometimes, they are not able to provide me the right atmosphere for work. Just working at home has affected me a lot, and sometimes I get busy with some guests and family, and at the same time my manager calls me asking me for a specific task to fulfill. This affects me and causes a big tension on me. Often when I go to my family house, I take my laptop or my cell phone with me because I might need it to work there. Despite all of this, we were implementing what was asked of us, but at the expense of my time, my children and my home”. RSS_Jabalia 2.*

*“We were doing our job online via the Internet despite the frequent internet cuts, and it was very difficult for us”. RSS- Rafah 4.*

### Theme 3: determinants with bi-directional relationship with COVID-19

Almost all participants, UNRWA staff and community members alike, mentioned that the Palestinians in Gaza were heavily affected economically during the pandemic. The additional expenses that UNRWA endured due to the pandemic stretched its limited resources and threatened staff’s salaries.

*“Recently, the Director of UNRWA operations in Gaza, said two days ago, that they will give the staff half a salary next month, due to UNRWA’s limited resources. He also requested the support of donors and European countries to enable UNRWA to continue providing its services to citizens.” Nurse_Rafah 7.*

The Palestinian community in general suffered economically as many people in Gaza rely on daily-pay. This has pushed Palestinians to break the lockdown rules in order to go to work and provide to their families. Due to the economic hardship, people had to prioritise spending, favouring buying food over face masks, for instance. Such practices increased the stress level amongst people as they struggled to make ends meet and they knew they were susceptible to contract and/or spread the virus.

*“Many people will die of hunger if they do not go to work. For example, if there is closure in Gaza including the markets, people cannot stay at home, as they want to work and gain money for their families. As a result, people break the lockdown measures because it is difficult to stay at home, and people don’t have any other sources of income but their jobs… The economic situation is very difficult, some people do not have the price of the face masks, and there are some people who are taking antidepressants and antipsychotic medications to get through this period” Community member_Jabalia 1.*

Unemployment rate was already high in Gaza prior to the pandemic. Many employers closed their businesses during the pandemic, increasing joblessness even further and adding to the psychological challenges that people were going through. Even at UNRWA, daily-paid staff were threatened to lose their jobs or go through extended periods of reduced income especially at the beginning of the pandemic when services got disrupted and before allocating these staff into new tasks. For example, daily-paid staff were allocated later on to help in food parcel distribution and medicine home-delivery; both of which responses were implemented later on into the pandemic.

*“Yes, sure, it [employment] was greatly affected, and it is known that unemployment is high. The situation during the pandemic is abnormal, and the general situation got worse, as many factories and shops have closed and suspended the work of their employees. This actually has worsened the psychological wellbeing of people as unemployment rate increased. “RSS_Jabalia 3.*

*“Gaza was significantly affected by UNRWA’s economic crisis… This also has affected the staff efficiency, in a way or another because there is no job security…. the daily-paid staff was affected in the beginning of the pandemic, as some of them have been laid off”. RSS- Rafah 5.*

### Theme 4: UNRWA’s response strategies

Interview data showed that, during the pandemic, UNRWA-Gaza deployed response strategies to counteract the impact of some of the social, economic and health-related stressors. With a focus on preventing COVID-19 transmission, UNRWA maintained access to health services by reconfiguring service provision; establishing medical points and introducing telemedicine services. Vulnerable groups, at risk of contracting the virus and with higher likelihood of displaying severe COVID-19 symptoms, were protected even further, as UNRWA introduced flexible working schedules for staff and introduced home-delivery of life-saving medications to prevent interruption of treatment. Patients with mental health illnesses were prioritised; allowing face-to-face interventions for some cases and establishing a hotline service. At the economic level, UNRWA rearranged the method of food parcel distribution in a way that protected staff and people from contracting the virus. Participants reported mixed views around all these response strategies. In general, interviewees said that strategies were generally effective yet, not enough to protect all segments of the society and meet their needs.

f) Enhancing safe access to health care

Early on in the pandemic, UNRWA Gaza revised its mode of service delivery to continue providing health care to its beneficiaries. Social distancing was ensured inside the health centres and stations for sterilizing the hands were put in place. Later on, UNRWA established a triage system whereby schools were converted into medical points that accepted patients presenting to the clinic with respiratory symptoms. Interviewed UNRWA staff and community members reported positive feedback, saying that community members showed a great deal of acceptance of this new mode of service delivery.

UNRWA introduced a telemedicine service in order to limit foot traffic at health centres, thereby limiting the spread of the virus amongst community members and staff. One community member from Jabalia said that UNRWA provided the Clinic Friend’s Committee with phone numbers and names of physicians that the committee in turn distributed to community members. Patients could call, for free, UNRWA health centres and receive consultation over the phone and then collect the medicine from the health centre later on. Those that required urgent medical attention were also visited at home by emergency teams.

*“…for the treatment of children or adults, there was a free number that they [UNRWA] gave it to everyone. In this regard, we held a meeting in the committee and we took these free numbers and sent them to social media and every other means that can reach patients, so that people know that there is a free number for the clinic, and through this number a person can call UNRWA and tell them about their illness and then provide them with treatment. Names of doctors were also listed to be in contact with people, and each doctor goes to the patient. Of course, this is for emergency cases”. Community member_Jabalia 1.*

The same participant however said that people complained that no one was answering their calls or the line was busy most of the time. Another participant from the same camp had the impression that the service provided by the emergency teams did not last for long.

*“At the beginning, the visit to the patient’s house operated for a week after that UNRWA stopped this method. UNRWA was not at the level required. UNRWA is expected to provide support and relief greater than that.” Community member_Jabalia 2.*

Another participant from the UNRWA clinic’s friends committee had the perception that the majority of community members preferred seeing their care provider in person over a remote service.

Providing health services to all segments of the society seemed to be disrupted for at least a month after the community transmission of the virus in late August 2020. A psychosocial worker from Jabalia camp said that she received complaints from patients for not receiving proper health care. Particularly during the transmission period, UNRWA prioritized NCD patients over other patient categories labelled as less urgent cases.

*“Because my work involves communicating with people, I receive several complaints. You know, patients are not limited to NCDs and pregnant women; we have many patients with other diseases who must come to the clinic to get the necessary treatment. In my opinion, I think it would have been better, if we applied work shifts, three shifts instead of two, to enable all patients to come to the clinic to get their treatment or to enable all patients, not just NCDs patients to access the telemedicine service, like patients who need general medications, like ointments and flu medicines. There is also a category requires care, like those who need to change wound or surgical dressings.” Psychosocial counselor_Jabalia4.*

g) Protecting vulnerable groups

Vulnerable groups, known to be at a greater risk of severe and fatal COVID-19 disease, such as older adults and patients with chronic conditions, were supported by UNRWA. Measures to prevent both groups from contracting the virus were put in place, thus lessening the stress level experienced amongst these groups; For example, staff members who are either elderly with respiratory disease and/or have NCDs had the freedom to work from home during the community transmission period of the virus.

*“(during community transmission) UNRWA was very flexible with both staff with chronic diseases such as NCDs and elderly staff members. These two groups can stay home, if they want to” Nurse_Rafah 7.*

Patients with NCDs were asked to stay at home and had their medications delivered to their place of residence instead of attending in person to the health centres, not only to protect them from contracting the virus, but to ensure that there was no interruption of treatment.

*“UNRWA was very supportive during the pandemic. I am only 45 years old and UNRWA started delivering my medication for hypertension ever since the pandemic started. UNRWA staff were putting themselves under risk in order to deliver medicine to us at home. The delivery was prompt and everyone received his medications on time” Community member_ Jabalia 1.*

*“They [NCD patients] stopped attending the clinic on the 24th of August. Before this period, not all NCD patients can come to the clinic, only those aged 42 or below and who are in a good health. Currently, we do not allow any of them to attend the clinic again, alternatively we are following them up over the phone… Also we are delivering medication packages to NCD patients’ homes in order to reduce the number of patients coming to the clinic.” Psychosocial counselor_Jabalia 4.*

It was reported however that the care pathway for NCD patients was disrupted during the pandemic. Exit permits outside the Strip became more difficult to obtain and Gazans with chronic conditions, mainly cancer patients, who are in need of medical treatments outside the Strip, faced dramatic limitations in accessing proper care.

*“In terms of health, some patients have been affected, like cancer patients who want to get treatment outside Gaza, for example in Jerusalem, and that requires complex coordination with the concerned authorities” Nurse_Rafah 7.*

Medical visits became less frequent due to UNRWA’s scaling down of non-urgent visits. Also, primary and secondary preventive measures of NCDs were halted. This disruption in the care process suggests a potential worsening of the chronic conditions along with the mental wellbeing of the patients.

*“…In general, we suspended all the regular medical tests for patients with chronic diseases.” Nurse_Rafah 7*

h) Improvising a new method for the delivery of food parcels

UNRWA Relief and Social Services Department usually provides food parcels for registered refugees who qualify according to a certain vulnerability checklist. During the pandemic, UNRWA continued to distribute food parcels but had to improvise a new distribution method that involves minimum contact in order to protect the community as well as the staff. In order to do that, emergency teams were formed that included staff working in different departments. The reconfiguration of the distribution process incurred delays and that negatively affected the people who urgently needed such assistance. The perception of delay in distributing food parcels was shared by UNRWA staff and community members.

*“There was also a delay in delivering subsidies (coupons every 3 months) to people for certain reason, as I got to know. Accordingly, this was a problem for people, who depend heavily on such coupons… people live under very difficult circumstances”. RSS- Rafah 5.*

*“The biggest problem is the food basket (coupon). This assistance has stopped for months, and some people are completely dependent on it…There is a delay in providing health services and food aid.” Community member_ Rafah 2.*

A community leader in Jabalia complained that some of the distributed food parcels were expired or close to their expiration date. Another community member who had over 25 years’ experience in volunteer work and was a member in Rafah said that the quantity of the food distributed was not enough; a view that was contradicted by another community leader in Jabalia, who is also a member of UNRWA Clinic Friends committee.

*“… UNRWA increased the quantities of food parcels and delivered medicines, and any request that people asked for from UNRWA was met. This is what I saw in reality.” Community member_ Jabalia 1*

i) Prioritizing mental health services

In general, all participants agreed that the pandemic was burdensome for Palestinians and had an adverse effect on their mental health. Protection and suicide cases were dealt with as emergency cases and were therefore granted permission to receive counselling face-to-face and were also referred to other departments or entities, depending on the severity of the case and the needs of the patient. The issue of specialists’ availability was voiced by few participants as a long-standing problem that dated from before the inception of the pandemic.

*“… at the beginning of the pandemic, Gaza registered a large number of [mental health] cases... we coordinated with doctors and triage nurses that urgent cases, such as suicide and protection cases must come to the clinic because they require direct intervention… Before the pandemic, unfortunately there were only one or two psychiatrists to deal with the cases having mental issues in all of UNRWA’s clinics in Gaza. This psychiatrist will come to the clinic once or twice a month. We receive many cases that require psychiatrist consultation and therefore, we requested to have at least one psychiatrist at the clinic. May be our doctors can prescribe psychotic medications, but some cases require the intervention of a psychiatrist.” Psychosocial counsellor_ Rafah 5.*

Prior to the pandemic, Palestinians in Gaza relied almost entirely on the services and aid provided by UNRWA as many residents are refugees themselves.

*“The percentage of refugees in Gaza Strip is very huge, and UNRWA represents the main pillar on which they depend on, especially in light of the conditions in which Gaza Strip lives such as: siege, poverty, power cuts, division and many problems.” Community member- Rafah 2.*

In the wake of the pandemic, UNRWA has done the best it can, utilizing its limited resources, to meet the increased needs, including mental health needs, of Gazans.

*“We have circulated our numbers, so that refugees can contact us whenever they need a consultation or help in this regard [MHPSS]. We divided Gaza into five areas; North Gaza, city of Gaza, middle of Gaza, old city of Gaza and Rafah and for each area, there is a psychosocial counsellor, so any refugee has any inquiry or concerns can contact them”. Head of Health Center_ Rafah 1.*

*“We received many protection and violence cases in June and July and we have no time to handle all of them.” Psychosocial counselor_Jabalia 4.*

Both psychosocial counsellors in Rafah and Jabalia camps mentioned that their work scope included providing counselling and awareness to patients presenting with respiratory symptoms (including those identified as infected with COVID-19), to NCD patients, and to other beneficiaries who visited the clinic up until the period of virus transmission, when all services became delivered over the phone. Staff support was also provided by psychosocial counsellors.

*“… You know, during the lockdown, all the family members were staying at home and this definitely had increased family problems, violence, GBV and suicides… The psychosocial workers also started in March to provide support and conduct self-care and stress relieving sessions for all staff members including cleaners and clerks”. Gaza Field Office staff 5.*

UNRWA’s response in Gaza, however, was not enough to alleviate the economic and social instability. Certain population groups were perceived to have been more acutely disadvantaged by the pandemic more than others. Increased violence, anxieties and worries were observed amongst women, children and workers in the informal sector and their mental health needs were not entirely met.

*“… I say we’re relatively limited in what we can do, you’ve got too many people in Gaza with PTSD given what they’ve gone through in the last 20 years, so we have to be realistic about our ability. We weren’t able to meet the needs before this. So we’re less equipped to meet increased needs during it. They are massive needs and we do what we can, but we were under no illusions.” Headquarter staff 3.*

UNRWA adopted a hotline service for people to seek assistance during the crisis. That included a special hotline for GBV cases whereby women call and seek help. GVB victims are then referred to the Protection Department for further assistance.

*“… we have a hotline to provide social support and a hotline for GBV cases; women who are subjected to gender-based violence. We were receiving calls from people who exposed to gender-based violence and such cases were transferred to the protection department. We installed the hotline to enable people to contact us to get our guidance and help, even if we can’t reach them.” Headquarter staff_2.*

## Discussion

Recent evidence shows that the Coronavirus pandemic has halted for some time the advancement towards achieving the SDGs by 2030 [32, 33]. The UN Department of Economic and Social Affairs published a report the SDG Report 2020 during the UN’s 75th anniversary. In this report, the UN mentioned that the pandemic has attenuated the implementation towards many SDGs and worse, in some cases, it has turned back decades of progress. Populations most affected by the pandemic are world’s poorest and most vulnerable populations such as people in the informal economy, children, older people, people with disabilities, migrants and refugees [34].

Looking through the lens of social determinants of mental health (Fig. 1), the current study contributes to our understanding of the pandemic’s effect on refugees, notably in the exceptional setting of Gaza; a setting marked by a high and longstanding levels of instability at the political, social and economic fronts. In reference to the framework (Fig. 1), we notice that the pandemic and/or its corresponding protective measures exerted an adverse effect on mental health using multiple pathways. First, the pandemic prompted an interaction between different factors from different domains thus, contributing to ill mental health. For example, interview findings showed that the stay-at-home orders and closure of non-essential businesses increased financial hardships and led to increased levels of unemployment in Gaza, which in turn, increased social disparities and hindered people’s ability to provide the means for their children to access online schooling thus, adversely affecting the mental health of children and prompting actions of violence and aggressiveness amongst school-aged children. Second, the pandemic exerted a direct impact on social determinants in all domains leading to adverse consequences on mental health. For example, interview findings showed that the pandemic increased the incidence of GBV against women. Evidence from the literature showed that levels of GBV and domestic violence have increased in the occupied Palestinian territory during the pandemic [5, 35]. In November 2020, UNRWA reported an increase in Gender-Based violence (GBV) cases against women and girls during the pandemic, in all fields of operation. The severity of physical assaults and psychological abuse reported by women was found to be greater than usual and was found to be associated with lockdown measures and movement restrictions [36]. Another observation on the interaction between the pandemic and social determinants pertain to the pandemic’s effect on factors related to the Social and Cultural domain (Fig. 1). Social cohesion, the sense of shared identity and the support received from the social circle are elements that are usually found in the Gazan community and Gazans often draw upon when facing and managing threats [37]. In the current study, the presence of a supportive work environment, reported by some of the UNRWA staff, and the act of giving help to fellow community members, reported by some UNRWA staff and community members, suggests that the presence of such a positive social support could have positive psychological consequences [38]. However, the nature of the threat that imposed social and physical distancing eroded to a great extent the social cohesion and support that Gazans usually rely on during crisis. Stigma and social isolation, primarily experienced by the COVID-19 survivors and their families, suggests a negative impact on the physical, mental, and emotional wellbeing amongst this group [39, 40] and could worsen the psychological wellbeing especially for those who had already mental health difficulties prior to the pandemic. A recent study in occupied Palestine found that, in a social distancing era, close social support becomes vital for mental health and exerts a stronger contribution to positive wellbeing than the general sense of belonging to a community and trust in government [41].

Refugee communities worldwide experienced stressors similar to those experienced by refugees in Gaza, such as increased GBV, financial strain, food insecurity, and poor access to education and were all found to exert a negative impact on mental health during the pandemic [42–45]. Protective measures, such as physical distancing, were found to be very challenging, if not impossible, for refugees living in different encampments across the world [46]. For Gazans, physical distancing was practically impossible, especially those living in overly populated camps. According to the WHO head of office for the occupied Palestinian territory (oPt), Gaza remains a very challenging environment making a variety of measures to curb the spread of the COVID-19 virus were unattainable in Gaza. This is due to over-crowdedness and shortages in everything, including electricity, medications and other supplies [47]. Also, the poor socioeconomic status of the majority of Gazans and their reliance on daily pay mostly made the enforcement of long durations of lockdown very challenging and unacceptable by the community. The proximity of households, lack of resources, including financial resources, and lack of jobs and savings were challenges identified in another study on internally displaced populations (IDPs) of Mali. Unlike in Gaza, the government of Mali, with the help of other humanitarian actors, responded by providing financial aid and creating some income-generating activities [48]. The absence of an appropriate mechanism to ease the economic pressure on Gazans was particularly burdensome and fuelled a climate of uncertainty. As a result, lockdown measures in Gaza were met with protests [5].

The Israeli forces seized the opportunity of the pandemic to tighten the blockade even further. This has further contributed to the worsening of the economic situation in Gaza and undermined the living condition of Gaza’s 2 million residents. Between March 2020–September 2021, Israel imposed a “Coronavirus closure” at the Erez Crossing, the only crossing point between Israel and Gaza, thus restricting the movement of people and goods even further. Except for a small number of patients in need for a critical medical treatment, movement of people was completely halted including those who travel for work-related purposes [49]. The same report mentioned that by March 2021, the number of people granted permission to cross the Erez Crossing did not exceed 6% of what was allowed the year before. Previous data published by the WHO indicated that about 9000 patients annually need to obtain Israeli exit permits to receive medical treatments outside the Strip, a quarter of this number is allocated to cancer patients [50]. Obstructing the freedom of movement for patients impedes access to proper health care and poses devastating consequences on patients with chronic conditions, especially cancer patients, and on the general health and wellbeing of Gaza residents [51].

Findings from this study were in agreement with those published Hammoudeh et al. [5]. In particular, women, children and daily workers were reported to be the most disadvantaged population groups. The increased financial strain, violence especially GBV, food insecurity and other stressors that were secondary to the pandemic serve as a stark reminder of the need to broaden the public health response beyond disease prevention to include social and economic interventions to improve people’s mental health and to decrease their vulnerability to future shocks. The fact that mental health disorders are socially determined means that improving access to mental health services without addressing the social determinants behind mental illness will not reduce the global burden of these disorders. Therefore, we recommend the implementation of multi-sectoral strategies and provision of comprehensive primary care that address social determinants. At the level of UNRWA, further collaboration between the Health Programme and the Relief and Social Services Programme is encouraged. For instance, including social workers in mental health training, taking place at the Health Programme, will enable social workers in identifying people with increased mental health needs and refer them back to the health centers for treatment. Another example would be to develop a brief, validated screening instrument for social determinants of mental health and incorporating it into patients’ medical records. This would help health professionals in looking further upstream on the conditions that predispose individuals to mental health conditions and take action long before they occur [52]. Assessment of the social determinants, particularly during times of prolonged crises and emergencies such as COVID-19 pandemic, will aid in the identification of beneficiaries that have become entitled to receive assistance. Finally, UNRWA is encouraged to establish partnerships with local agencies and community groups to implement social prescribing. Social prescribing is another strategy that is found to be an effective method for addressing the social determinants of mental health [52]. Social prescribing encourages individuals to take responsibility for their health and well-being by promoting people’s active participation in their local communities and statutory agencies for practical and emotional assistance. Being a member of a community group and receiving peer support reduces emotions of loneliness and anxiety. Therefore, social prescribing helps people to be more physically active, improves their mental health, improves their quality of life and lessens the burden of chronic diseases [53]. It assists individuals in discovering a new sense of purpose by engaging in activities they may not have tried before, such as the arts, cultural activities, walking, running, gardening, singing, etc. [54]. Research evidence reveals that social prescribing is also beneficial for the health care system as it reduces a person’s GP consultations by an average of 28% [55].

UNRWA’s lack of adequate financial support by the international donors posed a new challenge to continue providing essential services, while the refugee needs increased simultaneously due to the pandemic. This has led to the disruption of GBV services, for example, between March and April 2020 [36]. During the pandemic, UNRWA resorted to telemedicine to maintain patient care, reduce foot traffic at the health centres and the subsequent exposure to the virus, and to ensure continuation of treatment. Evidence in favour of telemedicine to manage chronic diseases isn’t conclusive. A global survey, addressed to 202 health professional in 47 countries, to evaluate the effect of COVID-19 pandemic on the routine care for chronic diseases found that diabetes, chronic obstructive pulmonary disease and hypertension patients were the most affected by the reduction in face-to-face care and inability to manage risk factors especially when morbidities co-exist [56]. Stronger evidence from studies conducted worldwide showed that the use of telemedicine could be helpful in patients’ assessment, disease diagnosis and treatment. There is evidence that patients with diabetes, cancer, transplanted kidney and those who needed prenatal care were managed using telemedicine and patients reported general satisfaction using this mode of service delivery [57–60]. As such, telemedicine has the opportunity to provide continuous follow-up care for NCD patients and other patients, whether during a pandemic or not. However, it remains important to prioritise outpatient visits based on disease severity in order to avoid non-COVID-19-related mortality and morbidity [61, 62]. Some barriers related to patients’ privacy and quality of care have been reported. As a result, more research is needed to assess this method in terms of its efficacy and quality of care [63]. Another concern is the fact that the use of such mode of service delivery could be impractical or not regularly feasible in settings that lack resources and/or have weak infrastructure [64]. Finally, telehealth is useful in overcoming barriers to access such as transportation and time spent commuting yet, it can be intimidating and confusing for older adults and other marginalized communities such as low-income households and people with disabilities [65]. As pointed out by Tran et al., telemedicine is not merely the transition from face-to-face to virtual service delivery. A successful deployment of telemedicine requires having health professionals who are trained at the personal and professional level to provide virtual care [66, 67].

In the case of mental health services, UNRWA restricted health visits to emergency situations only and resorted mainly to mental telehealth services and counselling over the phone for people reporting mental distress and for COVID-19 infected individuals. Evidence has shown that refugee populations, people with poor socio-economic status and those with previously diagnosed PTSD and depression have an increased risk for mental disorders during the pandemic, including the worsening of PTSD and depression [68, 69]. Eighty percent of health professionals in the global survey, mentioned earlier, reported a decline in the mental health status of their NCD patients [56]. During the pandemic, teletherapy became popular and health systems, such as UNRWA, were required to revamp their modes of service provision and deploy teletherapy on a short notice. Adequate training and workflow efficiency are practical elements that need to be present, especially in the absence of a framework to delivering mental health care through teletherapy [70]. In the current study, interviewed psychosocial workers mentioned that remote mental health services, although is a great tool during emergencies yet, it cannot permanently replace face-to-face therapy. This is in agreement with other studies that showed that the absence of non-verbal communication between the therapist and the client in teletherapy limits the ability to connect, build rapport and establish trust [71]. Moreover, permanently replacing face-to-face therapy with remote therapy would add to the feelings of loneliness and isolation and deteriorate mental health even further [72]. One study that was conducted prior to the pandemic found that the use of a hybrid approach, a combination of an online and face-to-face therapy, improved the promptness by which patients could be seen (timeliness of care) and the likelihood patients attend outpatient visits [73]. Perhaps a similar model could be useful as a long-term solution.

Based on previous research conducted prior to the pandemic, a remote interdisciplinary approach that involved social workers and psychologists was found to be effective and helpful for people in distress [74, 75]. Since the current pandemic was shown to affect people’s mental and physical health needs and alter their social circumstances, policymakers and health care providers are urged to consider setting up and promoting programs that operate remotely based on existing collaborative care models [76]. Such programs are useful in addressing patients’ changing mental health needs while targeting their evolving medical and social circumstances.

### Strengths and limitations

First, the identified themes reflect the opinion of interviewed participants and cannot be generalized; however, the daily stressors and difficulties encountered by Palestine refugees in this study were very similar to the challenges encountered by other refugee communities elsewhere. Therefore, the results of our study could be transferable to other contexts with similar characteristics. Second, interviews were conducted remotely as travel restrictions were put in place in order to minimize the transmission of the Virus. It is well-known that building rapport with participants is an essential component for a successful interview and a delicate process whereby body language and non-verbal cues are important. For many participants, however, remote interviewing provided them with a space to express themselves, the personal stressors they were going through and disclose their emotions. Third, the poor internet infrastructure in Gaza often resulted in interruptions during the interviews. All interviews that were interrupted were rescheduled to another day so that all consenting interviewees were given the chance to participate. Finally, interviews were carried out either via MS Teams or as WhatsApp calls. Allowing the candidate to choose to connect via diverse mediums enhanced recruitment and made participation possible for those with low computer literacy.

## Conclusion

Palestinians have historically lived lives of hardship- surviving in a context of occupation, chronic adversity, lack of resources and limited infrastructure. Similar to other refugee communities, the impact of the pandemic and its corresponding public health responses on Gazan’s health and daily life was significant. Due to scarce resources, the Gazan community had fewer means to withstand the economic and social repercussions of a pandemic. The pandemic served as a stark reminder of the need to broaden the public health response beyond disease prevention to address social determinants in order to improve people’s health and decrease their vulnerability to future shocks. The implementation of public health measures, such as extended periods of lockdown and physical distancing, were economically and socially not feasible. Strategies enacted by UNRWA, such as the triage system and home-delivery of medications, were welcomed by the community. Food support, in-kind assistance, access to online education were inadequately provided to protect all segments of the society. Telemedicine and mental tele-therapy need to be evaluated if they were to continue being adopted post-pandemic and/or for better preparation for a similar crisis; the use of a hybrid approach, a combination of an online and face-to-face therapy, could be useful as a long-term solution.

## Supplementary Information

**Additional file 1.**

## Data Availability

Interview recordings and transcriptions that were collected and analysed during the current study are not publicly available in order to protect respondents’ confidentiality and to safeguard personal or sensitive information that the respondents might not want to publicly disclose. Data are however available from the corresponding author upon reasonable request and with permission of UNRWA HQ.

## Abbreviations

CRIES-13: Children’s Revised Impact of Events Scale-13
GBV: Gender-based violence
HQ: Headquarters
IDPs: Internally displaced populations
MHPSS: Mental health and psycho-social support
NCDs: Non-communicable diseases
oPt: occupied Palestinian territory
PTSD: Post-traumatic stress disorder
QMU: Queen Margaret University
SDGs: Sustainable Development Goals
SRM: Security and Risk Management
UNHCR: The United Nations High Commissioner for Refugees
UNRWA: The United Nations for the Relief and Works Agency in the Near East
