# Supplementary material for: Social determinants and mental health needs of Palestine refugees and UNRWA responses in Gaza during the COVID-19 pandemic: a qualitative assessment
Source: BMC Public Health. 2022 Dec 8;22:2296. doi: 10.1186/s12889-022-14771-9 (PMC9733234; doi:10.1186/s12889-022-14771-9)
Supplement: Supplementary file 1 — Additional file 1. [file 12889_2022_14771_MOESM1_ESM.docx]

Supplementary Material_1

### 1- Key informant topic guide – HQ and field office staff

Please can you briefly introduce yourself, and your current role within the organization.

*Prompts: occupation, how long have they been in this role, has this changed since COVID-19*

From your position, how did you perceive the emergence of COVID-19? How did you perceive UNRWA’s readiness to manage a potential outbreak?

*Prompts: ease of collecting and analysing information related to COVID-19 (centrally, field levels), ease of liaising with different departments within a specific UNRWA field (Gaza/Lebanon) to act promptly, the extent whereby UNRWA is able to anticipate and cope with unplanned events (e.g. escalation of number of cases within camps), the extent whereby UNRWA is able to respond in an effective and acceptable manner, the ability to mobilize or move resources at short notice, the type and level of expertise needed to respond to the situation*, *any particular challenges affecting system readiness in Lebanon*

What steps did the organization take to respond to the COVID-19 pandemic?

*Prompts:*

*a) what public health measures were being considered (introducing shielding measures or infection, prevention and control) and what were the debates and decisions surrounding these? What steps were taken to ensure implementation?*

*b) for each of the steps, how were decisions made and who was involved, what expertise did these persons bring, what was the relative timeline of steps,*

*c) are emergency plans in place: what types of emergencies are covered, how have they been developed and updated, by whom, who fed into that process, have they been used for COVID response, what data is used;*

*d) what financial and other resources does UNRWA rely on in emergencies, how are these secured, what is the role of other local authorities and NGOs in securing these*

*e) how is communication with communities taking place, how is communication between the different levels of UNRWA taking place,*

*f) what values guides the steps of this response? And in general emergency response and delivery of services?*

*g) is the economic crisis in Lebanon affecting your response?*

What is the role of the COVID-19 coordination body?

*Prompts: how was the coordination body established, why, what is its role and remit, what communication pathways and methods does it use, who does the coordination body respond to, how does the organization (or the coordination body) receive feedback from patients or community members*

What policies have been developed in relation to the COVID-19 pandemic? What has informed the development of the policies?

*Prompts: link to other international policies (e.g. WHO, World Bank, UN Agencies) and national policies, do policies differ by setting, what is applicability across settings, do specific policies exist for specific population groups (e.g. elderly, those with pre-existing conditions),*

Given the link between severe COVID presentation and NCDs, what are your institutional priorities in relation to NCDs? Think about both current challenges and potential preparation for future outbreaks.

*Prompts: How do COVID-19 policies link to policies and services on NCDs including management and prevention (from preventing and managing risk factors, to managing onset of disease, to prevention and management of complications), what kind of policies are these, what stakeholders do they involve (prompt around multi-sectoral approaches), are there implementation barriers to prevention and what opportunities exist*

Given the link between COVID presentation and MH issues for individuals and families, what are your institutional priorities in relation to MHPSS? Think about both current challenges and potential preparation for future outbreaks.

*Prompts: how do COVID-19 policies link to policies and services on MHPSS, including management and prevention (from preventing and managing risk factors, to managing onset of disease), what kind of policies are these, what stakeholders do they involve (prompt around multi-sectoral approaches), are there implementation barriers to prevention and what opportunities exist (e.g. to prepare the service and the population fur future outbreaks)*

For field office staff: What is your relationship with UNRWA HQ? What is your relationship with local authorities?

*Prompts: what are the communication mechanisms, how frequent is communication, who is information shared with, what kind of information is shared, how is the public and community involved in communications and decisions, if additional resources are required how do you secure these, if new measures need to be introduced who would have the power to advocate for these or implement them, what influences whether or not decisions are adopted by the wider UNRWA system, how do UNRWA or local systems cater to persons with intersecting vulnerabilities (e.g. due to age, gender, disability), how are views from these latter groups considered when making public health decisions*

For Gaza: What local responses are being put in place in response to COVID-19? Comment on how this matches/not wider UNRWA response.

*Prompts: challenges to rollout of UNRWA response, how does the community perceive and respond to UNRWA*

For Lebanon: What is the national response to COVID-19? Comment on how this matches/not wider UNRWA response.

*Prompts: challenges to rollout of UNRWA response, how does the community perceive and respond to UNRWA*

How has the economic crisis in Lebanon impacted upon the spread and impact of COVID-19, and the measures UNRWA has introduced to manage this?

What are the challenges of preventing and managing COVID-19 in Lebanon given the ongoing economic crisis?

*Prompts: devaluation of currency and effect on resource availability, purchasing, aid, patient ability to secure medication and/or reach clinics*

### 2- Key informant topic guide – Health, social and relief worker topic guide

Please can you briefly introduce yourself, and your current role within the organization.

*Prompts: occupation, how long have they been in this role, do you feel heard in your role, what training opportunities and skills have you acquired, how inclusive is the workplace, has anything relating to your role or work changed since COVID-19*

*[In Lebanon: has anything in your role changed due to economic crisis]*

From your position, how did you perceive the emergence of COVID-19? How did you perceive UNRWA’s readiness to manage a potential outbreak?

*Prompts: ease of collecting, analyzing, and/or receiving up-to-date information related to available resources and to COVID-19 from HQ and field office, ease of liaising with different departments within a specific UNRWA field (Gaza/Lebanon) to act promptly, the extent whereby UNRWA is able to anticipate and cope with unplanned events (e.g. escalation of number of cases within camps), the extent whereby UNRWA is able to respond in a contextually accepted manner;*

What steps did the organization take to respond to the COVID-19 pandemic?

*Prompts: guidance and guidelines, communication with providers and with communities, measures to stop transmission (e.g. infection control, WASH)*

*[For Lebanon]:* How has the economic crisis in Lebanon impacted upon the spread and impact of COVID-19, and the measures UNRWA has introduced to manage this?

How have communities in your camp been affected by COVID-19 and/or the measures that have been introduced to address the pandemic?

*Prompts: food security, housing, crowdedness, education, social and family relations, employment, violence, how do UNRWA or local systems cater to persons with intersecting vulnerabilities (e.g. due to age, gender, disability), how are views from these latter groups considered when making public health decisions*

How do you feel about the current response to the COVID-19 pandemic?

*Prompt: the role of UNRWA within this, trust in the measures and messages, acceptability of measures, ability and willingness to act on public health advice, are communications clear, what is the local understanding of COVID-19 and enacted response by UNRWA*

Thinking about your current work, what services have been affected by the above measures?

*Prompts: access to the population, interaction with them, service continuity, quality of care, priority and allocation of resources, communication and relationship with patients, effects on health outcomes*

*Reminder: as possible, focus on tracer conditions (MCH, NCD) and also vulnerable groups, prompt consideration of gender (e.g. do you feel the experiences of female and male NCD patients differs, why?) or other factors (e.g. what challenges exist for persons with physical and/or cognitive disabilities)*

Thinking about your current work, how would you describe the availability of resources in the context of COVID-19?

*Prompts: PPE, sanitation, medications, equipment, finances, monetary and food assistance for the community*

*[For Lebanon]* What are the challenges of preventing and managing COVID-19 in Lebanon given the ongoing economic crisis?

*Prompts: devaluation of currency and effect on resource availability, purchasing, aid, patient ability to secure medication and/or reach clinics*

What challenges and stressors do you face in your role in the current time?

*Prompts: professional life (duties, workload, support, supervision, training, relationships with colleagues, personal mental health), personal life and relationship with work, relationship with patients, recent challenges in Lebanon due to economic crisis*

Thinking on the stressors that you have mentioned, how are you coping?

*Prompts: alternative sources of income, food security and health, family and social networks, sources of identity (religious, Palestinian)*

### 3- Key informant topic guide – Community leaders

Please can you briefly introduce yourself, and your current role within the community.

*Prompts: occupation, how long have they been in this role, organizations they work with*

What constitutes local leadership in this community?

*Prompts: experience, education, skills that make for a leader, how is someone recognized as a leader and by which groups*

What is your connection with UNRWA, if at all?

*Prompts: have they worked together or collaborated/received services, perceptions of the organization and its role within the Palestine community, trust in the organization – for what, when?*

In your view what does UNRWA represent for the Palestinian community?

*Prompt: right of return, current support*

How do you feel about the current response to the COVID-19 pandemic?

*Prompt: the role of UNRWA within this, trust in the measures and messages, acceptability of measures, ability and willingness to act on public health advice, are communications clear, what is the local understanding of COVID-19 and enacted response*

How are community leaders involved in the COVID-19 response?

*Prompts: trust in the measures and messages, acceptability of measures, are communications clear, perceptions of overall coordination between national and UNRWA and other organizations*

*[In Lebanon] How do you feel about UNRWA’s response to the economic crisis in Lebanon? How does this link with the organization’s response on COVID?*

What kind of stressors are you facing in your daily life? How have they been shaped by this pandemic? In Lebanon, how have these been shaped by the economic crisis?

*Prompts: role within community and family, health (e.g. in relation to existence of any other conditions, being able to seek care), stressors in daily life (food security, education, occupation, income, housing, neighborhood and environment, social cohesion and networks), family health and wellbeing, community health and wellbeing, conflict within local communities*

Thinking on the stressors that you have mentioned, how are you coping?

*Prompts: alternative sources of income, food security and health, family and social networks, sources of identity (religious, Palestinian)*
